# Deployment of Lidar from a Ground Platform: Customizing a Low-Cost, Information-Rich and User-Friendly Application for Field Phenomics Research

**DOI:** 10.3390/s19245358

**Published:** 2019-12-05

**Authors:** John T. Heun, Said Attalah, Andrew N. French, Kevin R. Lehner, John K. McKay, Jack L. Mullen, Michael J. Ottman, Pedro Andrade-Sanchez

**Affiliations:** 1 Maricopa Agricultural Center, University of Arizona, Maricopa, AZ 85138, USA; jheun@email.arizona.edu; 2 School of Plant Sciences, University of Arizona, Tucson, AZ 85721, USA; sattalah@email.arizona.edu (S.A.); mottman@email.arizona.edu (M.J.O.); 3 USDA-ARS US Arid-Land Agricultural Research Center, Maricopa, AZ 85138, USA; andrew.french@usda.gov; 4 Department of Bioagricultural Sciences & Pest Management, Colorado State University, Fort Collins, CO 80523, USA; kevin.lehner@colostate.edu (K.R.L.); john.mckay@colostate.edu (J.K.M.); jack.mullen@colostate.edu (J.L.M.); 5 Department of Biosystems Engineering, University of Arizona, Tucson, AZ 85721, USA

**Keywords:** lidar, phenotyping, maize, GWAS

## Abstract

Using sensors and electronic systems for characterization of plant traits provides valuable digital inputs to support complex analytical modeling in genetics research. In field applications, frequent sensor deployment enables the study of the dynamics of these traits and their interaction with the environment. This study focused on implementing lidar (light detection and ranging) technology to generate 2D displacement data at high spatial resolution and extract plant architectural parameters, namely canopy height and cover, in a diverse population of 252 maize (*Zea mays* L.) genotypes. A prime objective was to develop the mechanical and electrical subcomponents for field deployment from a ground vehicle. Data reduction approaches were implemented for efficient same-day post-processing to generate by-plot statistics. The lidar system was successfully deployed six times in a span of 42 days. Lidar data accuracy was validated through independent measurements in a subset of 75 experimental units. Manual and lidar-derived canopy height measurements were compared resulting in root mean square error (RMSE) = 0.068 m and r^2^ = 0.81. Subsequent genome-wide association study (GWAS) analyses for quantitative trait locus (QTL) identification and comparisons of genetic correlations and heritabilities for manual and lidar-based traits showed statistically significant associations. Low-cost, field-ready lidar of computational simplicity make possible timely phenotyping of diverse populations in multiple environments.

## 1. Introduction

The advent of sub-meter precision GNSS (Global Navigation Satellite Systems) hardware and ruggedized field-ready electronics has created an opportunity to develop a number of applications for field-based experimentation serving a variety of academic disciplines with interest in characterizing stresses in complex biological systems such as agriculture. Some of the benefits provided in this emerging field of the use of digital tools are increasing with improved rates of data acquisition and spatial resolution [1,2,3]. Moreover, these digital tools offer substantial improvements in repeatability and accuracy while maintaining their operational characteristics for prolonged periods of time. They also provide adaptability to various field and weather conditions.

Field-ready electronics are suitable for deployment from moving ground platforms, which are particularly useful in high-throughput phenotyping (HTP) applications of small-size plot research [3,4,5,6]. Engineered platforms provide off-road mobility as well as mechanical and electrical interface for electronics that capture and record data on-the-go. These instrumented platforms are capable of capturing a range of plant traits such as the thermal response of the crop to abiotic conditions, the spectral reaction to incoming light and reflectance, the architectural characteristics of growing plants, and other applications. This paper is focused on the implementation of displacement sensing technology aimed at characterizing the growth dynamics of maize plants with multiple frequent observations over time.

Canopy height and width are two plant characteristics of particular interest in this study. The height of maize plants at early phases of development is frequently defined as the distance from the soil to the uppermost leaf in the plant [7]. In this paper we report on canopy height estimations using a similar approach, which was defined as the distance from the soil surface to the highest point in the arc at the uppermost leaf whose tip is pointing down [8]. For maize plants approaching maturity stages, a common protocol uses the distance from the soil line of the plant to the base of the flag leaf [9], this measure excludes any variation in tassel length from the flag leaf to the top of the plant. This is an important consideration for sensor-based estimations because tassel length is difficult to estimate accurately given their much reduced size compared to leaves and stems. To characterize canopy width our approach was to compute canopy cover in the horizontal plane.

This manuscript describes with particular detail an HTP application of high-energy optical displacement sensing for canopy structure and size variables associated with plant height and width. Lidar (light detection and ranging) is a displacement measuring technology that uses laser (Light Amplification by Stimulated Emission of Radiation) light pulses. Lidar systems are powerful pieces of hardware that create large arrays of vector data. These point cloud data are massive in size and create technical difficulties in handling these data sets and makes the extraction of useful information very challenging. Chief among the challenges are the ability to rapidly and continuously log hundreds of megabytes (MB) to gigabytes (GB) of data generated at high rates, collecting and merging cm-accuracy geolocations, classifying the resulting point cloud data sets to plot levels, and rapidly summarizing plant geometry data. In this paper we will present the details of sensor integration to a ground platform, the data acquisition steps to record relevant data and reduce file size, the algorithms for post-processing, and the results of GWAS (genome-wide association study) analytical work performed on data sets generated with this lidar system.

High-throughput phenotyping measurements of canopy height and width in plant research are two important architectural parameters with intrinsic value in characterizing the growth and development of the crop. Plant height has been a breeding target due to its correlation to flowering, lodging, yield, and harvest index, as well as environmental factors such as nitrogen status [10,11,12]. The relatively high heritability of the trait makes it attractive for GWAS approaches. However the large number of genes affecting the trait [9] leads to high phenotyping needs.

The use of lidar technology in agriculture is increasing in popularity. Recent technological developments include precision agriculture applications such as biomass yield monitoring in specialty crops [13], plant spacing monitoring in vegetable row crops [14], and 3D modeling in orchard and vine crops [15,16], among others. Similarly, application of lidar technologies to field crops has been reported in research to study plant growth in cereal crops [17,18].

For the case of field phenomics research, turn-key lidar systems built for field applications have been implemented in a number of field research studies [2,7,18,19,20,21]. These systems are expensive and cost between $10,000 and $100,000+ USD. In most cases these systems are set up to generate very large data volumes, making it a computational challenge to extract information needed to feed the analytical models that follow the outdoor data collection phase. There is a need for easy-to-use lidar system deployment and mapping, and this paper reports on technical developments that fill that deficit. The following sections present technical details of the adaptation of low-cost industrial-grade lidar hardware originally developed for safety applications, along with customized hardware and software developed for successful field deployment and running of an efficient data pipeline. Among other benefits, with this lidar system we have achieved reliable operational capacity and outstanding performance in terms of throughput, data reduction and consistency.

## 2. Materials and Methods

### 2.1. Field Platform and Experimental Setup

**Figure 1:**
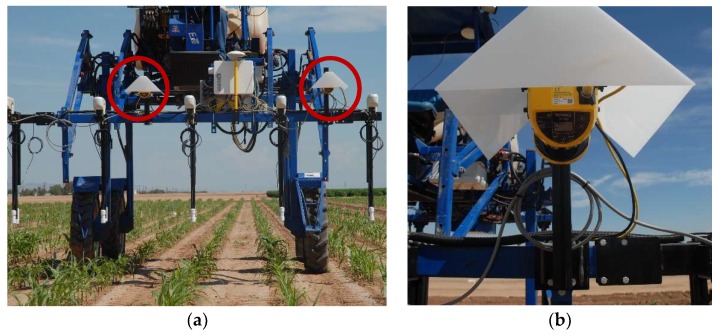
Instrumented ground platform showing electronic sensors and associated data collection hardware as deployed in maize field in Maricopa, AZ, USA. Circles in image (**a**) are the lidar systems positioned above center of experimental plots. Image (**b**) details the sensor mounting.

A pair of 2D scanning lasers were selected for high-throughput data collection that could cover four rows simultaneously. A custom-built electronic controller met the following criteria: robust operational characteristics to survive outdoor deployment in the high temperatures typically experienced in the low elevation deserts of Arizona; compatibility with available, rugged data logging equipment; ability to integrate into the existing electronic crop pheno-measurement system as a modular component (Figure 1); capacity to acquire a manageable but adequate amount of data; provides means to eventually process data in real-time after building functional algorithms to extract plant height, canopy width, canopy closure, and canopy density/biomass estimates. The dual lidar system and additional instrumentation was deployed six times during the 2018 Summer/Fall growing season from 17 August to 28 September 2018 on 22, 28, 34, 40, 47, and 64 DAP (days after planting).

The experimental layout consisted of 1728 experimental plots of 3.66 m by 1.52 m dimensions that were planted with two rows of 248 replicated accessions from the maize (*Zea mays* L.) SAM (shoot apical meristem) diversity panel [20] along with 4 NAM RILs (nested association mapping recombinant inbred lines) check varieties that included Z011E0187, Z013E0127, Z022E0104, and Z025E0085 planted 10 times per block, in three replicate blocks each of well-irrigated and drought-stressed treatments. The experiment was conducted at Maricopa, AZ, USA (33.0609725° N; 111.9697775° W) over a total area of 10,435 m^2^ of sandy loam soil and watered using flood irrigation. The crop rows were aligned to 0° north in UTM grid 12N using a power unit with GNSS RTK (real-time kinematics) navigation enabled. Seeds were planted using a Kincaid Voltra (Haven, KS, USA) 4-row precision planter designed for small plot research trials. Crop management followed accepted agronomic standards for maize production in the region.

### 2.2. Light Detection and Ranging (Lidar) Scanning System Development

#### 2.2.1. Sensor Specifications

The lidar sensors deployed were two of model# SZ-16D, made by Keyence of America (Itasca, IL, USA). The SZ-16D is a 2D scanning laser designed and certified for industrial safety applications with additional data output (2D scan measurements) functionality. The sensor has a Class I infrared laser diode with a 905 nm wavelength. The detectable angle/FOV (field of view) of the sensor is 270° with a fixed angular resolution of 0.36° between optical angles (270 ÷ 0.36 = 751 optical angles). Displacement measurements from each optical angle are represented in millimeters as a 14-bit value with a resolution of one millimeter. The scan frequency of the sensor is fixed at 32 Hz. The starting optical angle, total optical angles, and number of angles to skip are programmable. The sensor requires a 24 V power supply and typical power consumption is approximately 13 W for each sensor. The maximum operating temperature is 50 °C in a relative humidity range of 35% to 85% (non-condensing). The SZ-16D uses full duplex RS-422 communications send/receive data between the sensor unit and a computer or microcontroller. The maximum/maximum configurable baud rate is 9600/250 kbps, 8N1. The maximum data transmission distance is 10 m on battery power.

#### 2.2.2. Platform Installation and Sensor Alignment

**Figure 2:**
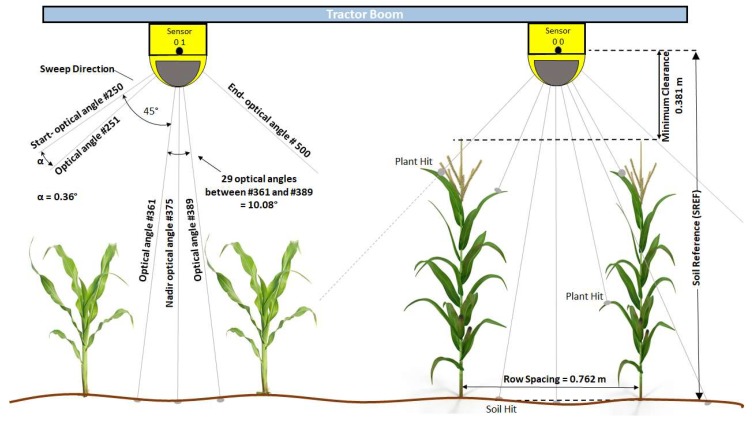
Two-dimensional (2D) schematic representation of dual light detection and ranging (lidar) units scanning two experimental plots, each consisting of two rows of maize plants.

Prior to the first field scan of the season, the sensors were added to the front-mounted tractor boom and fixed into position at 1.682 m above a level concrete floor using a combination of extruded aluminum T-slot bar, custom fabricated steel brackets, and adjustable bracket (Keyence part# OP-86938). The sensors were spaced 1.52 m apart, centered over a plot furrow to capture the soil between plot rows and acquire measurements from both plant rows within the plot (Figure 1 and Figure 2). An Astrodyne (Hackettstown, NJ, USA) 50 W, 24-volt direct current–direct current (DC–DC) converter (model# SD-50-24) was installed in a separate enclosure and connected to a 12-volt lead-acid deep-cycle marine battery for power. The power leads on the SZ-16 output cables (SZ-P5PM) were connected to the power supply through a switched relay.

A 2-m length of black anodized aluminum square bar was placed under the sensors and leveled to create a reference surface. A plumb bob was used to align the laser nadir axis with the reference bar on the concrete surface. Using the monitor function within the Keyence Safety Device Configurator software version 3.3.2.0 [22] installed in a laptop PC and connected via USB cable, each sensors’ sweep was checked for orthogonality with the leveled bar on the ground, one sensor at a time. The sensor mounting brackets were then adjusted to square the sensor with the reference bar on the ground (<0.05°) and equalize their vertical distance from the reference bar with an uncertainty of ± 5 mm.

Custom shrouds designed in-house were then attached to shade each sensor from direct sunlight (Figure 1b). The shrouds were made using 3.1 mm white acrylic and fabricated by Arizona Plastic LLC (Tempe, AZ, USA).

#### 2.2.3. Lidar Controller

A custom controller with multi-sensor networking capabilities was designed and prototyped in-house to configure the sensors, read and convert the sensor data into textual messages, and provide a foundation for developing higher levels of functionality. An Arduino Due board (Somerville, MA, USA) was used for the core of the controller and programmed using the Arduino IDE, version 1.8.3 (Somerville, MA, USA). A daughter card was prototyped to contain all the communications circuitry required to network multiple (up to four) laser sensors and provide output data in ASCII character form, structured similarly to GNSS NMEA (National Marine Electronics Association) messages, to a computer or, in this case, a CR1000X data-logger manufactured by Campbell Scientific (Logan, UT, USA) with a 100 MHz 32-bit processor running OS (operating system) CR100X.Std.01.02.

#### 2.2.4. System Networking: Sensor-Controller-Logger

The scanning lasers require a RS422 network to receive commands and send data. Details are found in the SZ-16D Communication Manual (1). A DS8921A differential line driver and receiver integrated circuit (IC) chip built by TI (Texas Instruments, Dallas, TX, USA) was used to provide the RS422 translation between the sensors and universal asynchronous receiver-transmitter (UART) UART1 (Channel 1) on the Due board. Pull-up and pull-down resistors were required on the receive lines to terminate the RS422 connection at the chip and provide stable line-biasing voltages. Two TI max232(x) RS232 chips were also installed to provide up to 4 serial I/O channels, but only two channels were implemented connecting to UART2 (Channel 2) and UART3 (Channel 3) on the Due motherboard. They were both used to configure the controller/sensors and output laser scan data to the CR1000X. The Arduino Due board utilizes the Atmel SAM3X8E CPU, which is a 32-bit ARM microcontroller clocked at 78 MHz and operates at the 3.3-volt logic level. All chips used were chosen to work with or adapted to the Due’s 3.3-volt level logic.

Each sensor’s RS422 port was configured at the maximum baud rate of 250 kbps, 8N1 and a matching rate set in the lidar controller programming. The two RS232 ports in the controller were configured to 115,200 bps, 8N1 because of the baud rate limit of the CR1000X RS232 ports and to remain compatible with standard PC baud rates for diagnostic ability. The sensors’ RS422 port terminal requires a Eurofast style M12-5 pin cable. A 5-m cable for each sensor networked them to the M12-5 pin terminals installed in the lidar controller enclosure. The controller was then mounted in a larger enclosure with the CR1000X data-logger. Shortened RS232 cables connected the controller to the CR1000X. This stand-alone system was then integrated into the existing electronic system, connecting a Trimble Ag552 RTK-GNSS receiver (Sunnyvale, CA, USA) (a third RS232 port) and two control ports on the CR1000X (C1 and C2) which link to the operator’s control remote. The GNSS positioning data was distributed to all data logging equipment by splitting (tee) the RS232 data to each device (CR1000X and existing CR3000). The two wires connecting the logger control ports served to synchronize the start and pause of data acquisition on all data-loggers. This strategy prevents collecting unnecessary data during turns at the ends of the field and further reduces data volumes by not recording information outside of the experiment boundaries.

#### 2.2.5. Sensor Optimization for Data Reduction

Optimization in timing becomes crucial in order to meet all operational needs/expectations with two networked sensors. The sensors work as slaves, performing measurements at a rate of 32 Hz. The lidar controller must call each sensor by their unique address and wait for a response. Since both sensors share the same communication lines (RS422), data transfer must be complete for one in order to start transfer from the other sensor. Once 2D sweep data is transferred from both sensor units, the controller processes the 2-byte binary values for each optical angle and translates them into a distance in millimeters. A custom message was formatted to be sent to the data-logger over a single RS232 port containing a header with the lidar unit’s unique address, a controller timestamp, some values for error checking, the average displacement of both sensors and soil measured during initialization, instantaneous displacement between sensor and soil, and each of the 251 optical angles in the sweep.

The time to acquire 251 data points from the dual unit network, process, formulate, and output the data messages using the highest baud rates on a single RS232 port was measured at 98 to 99 milliseconds for each message, for a total of 197–198 milliseconds (5.05 Hz) per cycle. The *millis()* function in Arduino C++ was used as a time stamp in the message outputs to measure the time between cycles during testing. Most of the cycle time was spent transmitting the message in convenient ASCII text with the point distances represented in up to four-digit decimal values of millimeters. The second RS232 port was then enabled to speed up the message output cycle so a timing function could be added to the controller firmware to control the output message rate more precisely and provide the option to configure the controller to set different output cycle frequencies. The two sensor messages are sent out interlaced between the two ports; each port delivers a full message of 251 data points from a single sensor. At full speed, this method only takes an additional 11 to 12 milliseconds more than the cycle time for a single sensor with a full cycle time of 109 to 110 milliseconds (9.09 Hz) for both sensors, leaving additional time for added functionality at the 5 Hz output rate. The header for lidar sensor unit 00 is passed to serial channel 2 then the header for lidar sensor 01 is passed to serial channel 3; the first optical angle for sensor 00 is passed back to channel 2, then the same optical angle for sensor 01 is passed to channel 3, and so on until the end of the messages.

**Figure 3:**
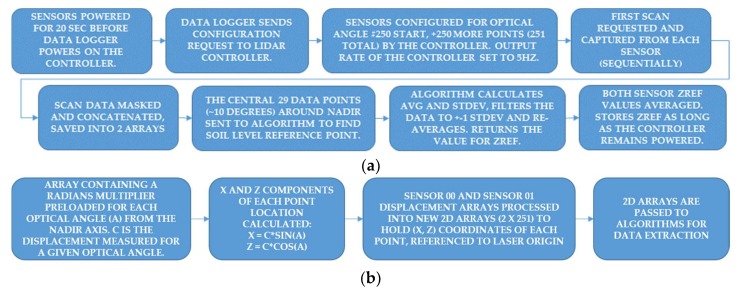
(**a**) Lidar system power-up and initialization procedure at the start of the field scan. The data logger configures the controller to auto-output sensor data at 5 Hz on 2 serial ports. The data logger captures sensor data from the lidar controller and Global Navigation Satellite System (GNSS) positions when the logger scan cycle is ‘un-paused’. (**b**) Example of coordinate calculation (x,z) for each point in the 2D laser sweep. The point (x,z) is in reference to the laser origin and nadir axis.

The SZ-16D scanning lasers have the ability to change the starting optical angle and number of following angles to output [16]. With data reduction and optimization in mind, the sensors were configured at each start-up by the lidar controller to reduce and fix the effective FOV of the sensor to 90° from the default of 270°. The starting optical angle was programmed to be 45° from the nadir optical angle. The total number of data points per laser sweep is reduced to 251 from 751 for each sensor, a 66% reduction in raw data points per sweep (1.278 KB from 3.778 KB). The mounting point of the laser units above the crop and soil then determines the optical angle lengths within the FOV and the resolution between points, which is simple to calculate using sine and cosine laws (Figure 3b). Figure 2 presents details on physical dimensions, radial resolution of lidar beams in scanning mode as implemented in the present study.

By design, data output frequency was targeted for 5 Hz from the controller to match the frequency of the measurements from the other sensors in the existing system. In having a similar number of total records as other sensors per field scan, data can be opened in a spreadsheet or text program, such as Excel, and be checked for quality within minutes of exiting the field.

#### 2.2.6. In-Field Sensor Initialization and Field Scan

A dynamic check that incorporated raw sensor data and processing algorithms was performed prior to field deployment. This test was carried out only once and before the start of the field season to quickly assess the overall system accuracy. With the sensor platform parked on a level concrete surface, the distance between the sensors’ origin (the origin is a reference mark that protrudes from the sensor housing) and the floor was measured to be 1682 mm using a tape measure. The system was then driven over a rough soil target area (about 30 cm in length along the path of travel) 3 times at the typical operational speed of 0.64 ms^−1^ and over compacted soil with natural roughness. The measurements were recorded with the data acquisition system and run through the post processor afterwards to confirm the system was performing at an expected overall accuracy of <25 mm. During the field season, a similar test was performed under static conditions inside the field during sensor initialization.

Initialization of the sensors is completed at the beginning of each field run. The lens covers of each sensor are wiped gently with a damp cloth to remove dust. The laser units are parked over a clear alley space between plots, in an area where the terrain is representative of the field conditions. This is done so that the lidar controller will configure the sensors at initialization, take a sample measurement using both sensors, and run a simple algorithm using the center 10° FOV (29 displacement values centered on the nadir optical angle), then take the average displacement of both sensors. This reference value is stored in the controller and reported in every message until the controller is powered off. Figure 3a is a block diagram showing the steps involved in sensor initialization at the start of the field run.

The lidar units require 20 s after power-up before configuration or data communications can initiate and so are powered up first. The data-logger controls power to the lidar controller and sends it a configuration request immediately after the logger program compiles. The controller repeats the configuration back to the logger and the bytes received by the logger are checked to verify the configuration was successful. The *Z*-axis reference scan (ZREF) is initiated right after the sent configuration confirmation and data begins to constantly flow from the controller until it is powered off at the end of the field run.

#### 2.2.7. Post-Processing and Parameter Extraction of Lidar Data

**Figure 4:**
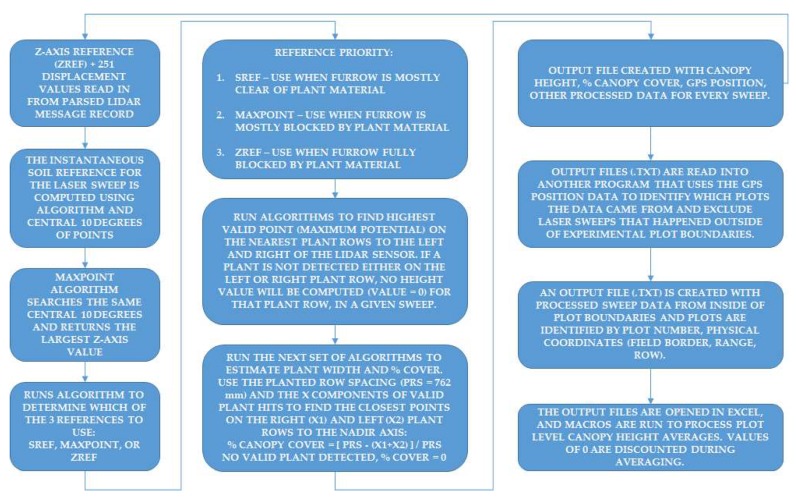
Post-processing flow of lidar point data into plot summaries per scan date. Each 2D laser sweep is processed for specific measurement parameters/products and written to a new file for each sensor. Those files are moved through the rest of the pipeline to create a summary file with plot averages and standard deviations.

The block diagram in Figure 4 provides computational details on the steps of post processing of the laser files into plot-level data. Two lidar record files (one for each sensor) are created from the CR1000X programming that contain a timestamp, record number, GNSS position information (parsed from the GNGGA and GNRMC messages), and the entire lidar message (unparsed). Each logger (.DAT) lidar sensor record file is reformatted so all of the optical angles are parsed from the text message, along with GNSS latitude and longitudes converted to degrees; latitude and longitudes are converted into UTM (Universal Transverse Mercator) coordinates and included as well.

The data processing pipeline consists of: a program written in C++ to read-in data files and run functional algorithms to extract parameters, such as plant canopy height and canopy cover then output a new file with these parameters and GNSS position information; a second program to read-in the extracted parameters output file, remove measurements that were collected outside of experimental plot boundaries (buffers plots and alleyways) and create a new file with each valid record tagged with the corresponding plot number and genotypic id/name; the new files containing plot-level data are opened into Excel and macros are run to summarize/average each plot. The two files with plots averages are then merged into a single text file containing all 1728 plot numbers. The time to post-process lidar files into plot summaries through the pipeline takes an average of 20 min to complete per field run.

### 2.3. Lidar Data Validation and Visualization

Lidar height and fractional cover estimates were validated using manual and photographic measurements. Manual measurements of canopy height were carried out in a selection of 75 experimental plots in coordination with the deployment of the ground platform on 25 September 2018 on 64 DAP. Lidar-based and manual measurements of canopy height were compared through simple linear regression to assess their degree of association at a time when morphological differences were at or near maximum. Using this set of 75 plot averages, root mean square error (RMSE) was computed between the manual and lidar-based estimations of canopy height.

The same set of 75 plots were then imaged for fractional cover estimation. Using an RGB Canon D5 camera fitted with an 8–12 m zoom fish-eye lens, images over individual plots were collected. The camera was installed on the end of hand-held pole, extended approximately 2.8 m above the ground, leveled and centered mid-plot. Use of the fish-eye ensured complete coverage of plots of interest with one image. Computations were based on image height, plot dimensions and an estimate of the image projection obtained using the equi-solid function defined below:

(1) $$r=2f\sin\frac{\theta }{2}$$

where *r* is the distance in the image of each pixel from the nadir pixel, *f* is the lens focal length, and *θ* is the view angle.

**Figure 5:**
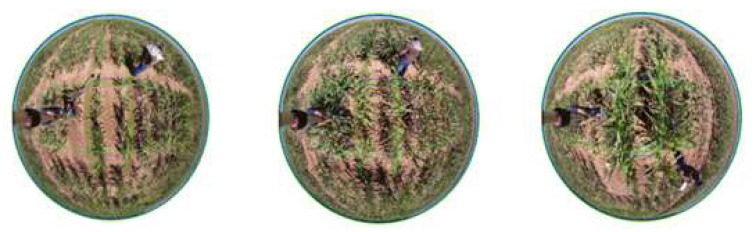
Overhead fish-eye images of plots 131 (**left**), 71 (**center**), and 139 (**right**) with genotypes of small, medium, and tall size plants respectively.

Each image was cropped to plot dimensions as indicated by the green colored lines in Figure 5. Cropping was done by defining plot-specific rectangular extents—nearly the same but not identical to the true curvilinear extent—with the R ‘raster’ library function ‘extent’, then applying the ‘crop’ function. Because of layover—distortion due to changing perspective away from the image principal point—image portions of tall maize plants sometimes fell outside of the rectangular extent and were cropped.

Initial classification was done using the trainable Weka Segmentation Tool plugin for ImageJ [23] with Fast Random Forest algorithm on RGB images. Samples of sunlit and shaded leaves and soil were created for training sets, then applied to the cropped images. Unfortunately, the routine was computationally slow, required large amounts of computer memory, sometimes crashing the OS (operating system), and had poor agreement with lidar estimates. In a second approach, each image was converted to a single-band normalized green vegetation index according to Equation (2), then classified according to its corresponding histogram.

(2) $$GNDVI=\frac{R_{green}-R_{red}}{R_{green}+R_{red}}$$

where *GNDVI* is green normalized difference vegetation index, *R_green_* is green-band reflectance, *R_red_* is red-band reflectance.

**Figure 6:**
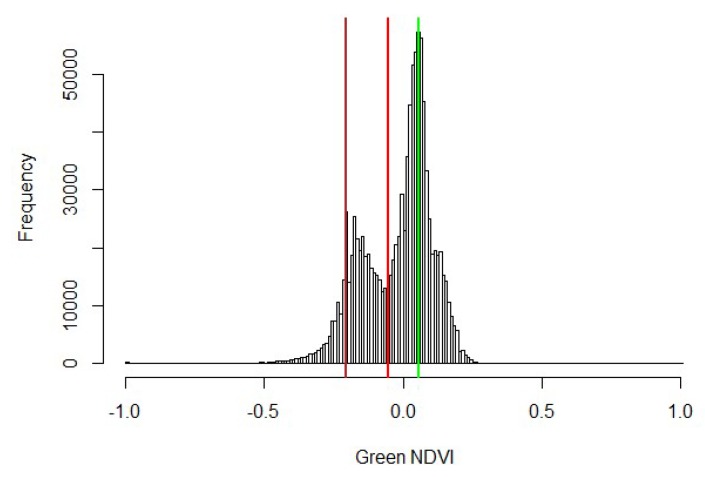
Typical green normalized difference vegetation index (*GNDVI*) histogram derived from overhead plot image.

At Maricopa the soil has a strong red component, meaning that the histograms had strong bi-modal patterns. Green plant fractions were counted as the ratio of pixel counts greater than the local minimum to the total pixel counts (Figure 6). Lidar-based estimations of canopy cover were compared to image-based estimations of fractional cover through simple linear regression.

The combination of operational parameters, lidar settings, and data handling resulted in each plot containing around 6000 lidar points. For lidar data visualization, first lidar points were extracted from individual plots, converted to PCD format, and then visualized using in-house R scripts running on software version x64 3.61. The open-source software package CloudCompare [24] version 2.11 (alpha) was used for confirmation.

### 2.4. Genetic Analyses of Geometric Parameters Derived from Lidar Data

We estimated breeding values using the least square means of each trait from mixed-model ANOVA in JMP Pro release 14 (SAS Institute, Cary, NC, USA), with genotype as random effect and row as fixed, split by treatment. Heritability was estimated as the proportion of the genotype variance component to total. The genetic correlation was measured as the Pearson coefficient for the breeding values. For genome-wide association (GWA) mapping, we used a mixed linear model (MLM) implemented in TASSEL 5, with a kinship matrix (K) and population structure (Q) as covariates [25]. Markers were from a previous study [26] and were filtered by minor allele frequency >0.05, resulting in greater than 860,000 single nucleotide polymorphisms (SNPs).

## 3. Results

### 3.1. Ground Platform Field Deployment and Lidar Data Generation

During the dynamic check, the SREF values (dynamic soil reference value used for plant height estimate in each 2D laser sweep) for sensor 00 were estimated by the lidar system (sensor and processing algorithms) and averaged 1675.8 mm, with a standard deviation of 4.7 mm, and the range of data points was 10 mm. Similarly, the average SREF values for sensor 01 over the target was 1684.1 mm with a standard deviation of 3 mm, and a 10 mm range of data points. These average SREF values showed a deviation of −6.2 and +2.1 mm for sensors 00 and 01, respectively, from the manually measured distance between the sensors and the soil surface (1682 mm) as described in Section 2.2.6.

The experimental field was scanned running 36 transects with no overlap between adjacent passes while the ground platform was operated at a forward speed of 0.48 m·s^−1^. Taking into account the platform speed of operation, total turning time outside the field, field size and shape characteristics, the platform performed at theoretical and actual machine field capacities of 87.7 and 73.0 m^2^·min^−1^, respectively. Therefore, the overall machine field efficiency of this ground platform was in the order of 83%. During the dates of field deployment, the lidar system generated information at an overall rate of 43.517 MB of text-based. DAT files per hour. This rate of data generation translated into 9.938 kB of lidar-based data per m^2^.

As described in Section 2.2.6, ZREF is an operational parameter that describes the sensor position in the vertical direction above ground level. The value of Z-REF changes every time the lidar system was deployed in the field. For field deployment on 22, 28, 34, 40, and 47 DAP, ZREF had an average value of 1.65 m (± 0.02 m). ZREF for 64 DAP was 2.23 m, a significant increase due to the high rate of growth that the tallest plants experienced.

Same-day in-house post-processing of collected lidar data yielded significant reductions in data volumes. The following points describe the sequence of steps performed, along with digital size of outputs and processing time during a typical field deployment day for lidar data collection:

1. Logger output text files (.DAT) in Compact Flash (CF) cards; 2.44 h of data collection = 106.2 MB
2. Reformatting for post-processor/output product extractor = 91. 5 MB → 10 min.
3. Processing output files (C++ program) = 7.587 MB → 3 min.
4. Run plot identifier and remove data from plot alleys (C++ program) = 5.907 MB → 25 s.
5. Run plot summary macros (MS Excel macro) = 316 kB → 10 s.

In steps 1–4 above, text-formatted output files for both lidar units combined contained upwards of 70,000 record lines with 251 delimited fields in each line. In contrast, output of step 5 was a. CSV file with 1728 record lines with by-plot statistics including mean and standard deviation of all lidar-based parameters. To illustrate the value of the algorithms involved in raw data postprocessing, Table 1 presents time-series data of three genotypes chosen for their differences in canopy size and overall morphology.

### 3.2. Validation of Lidar-Generated Canopy-Height and Canopy-Cover Parameters

**Figure 7:**
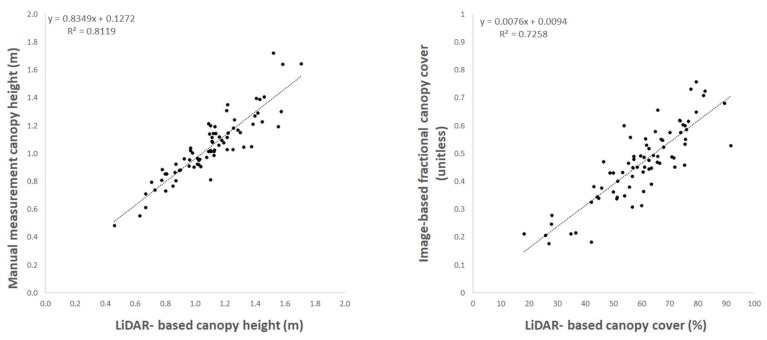
Linear regression plots of lidar-generated values and independent estimations of canopy height (**left**) and canopy cover (**right**) on a subset of 75 experimental maize plots.

The experimental site was composed of 1728 plots with two rows of maize plants each. The validation procedure is presented from a subset of 75 plots that were scanned with the ground platform on 28 September 2018 (64 DAP). The regression plots in Figure 7 show the degree of association between the lidar-based quantities and the corresponding values generated with independent methods of manual canopy height measurements and image processing of whole plot overhead images. The comparison between manual and lidar-based CH values yielded a root mean square error (RMSE) value of 0.12 m and the coefficient of determination (r^2^) in the regression analysis was 0.81. Similarly, the r^2^ value obtained with the regression analysis between lidar-based CC and the image-based fractional canopy cover was 0.73.

### 3.3. Visualization of Lidar Data

**Figure 8:**
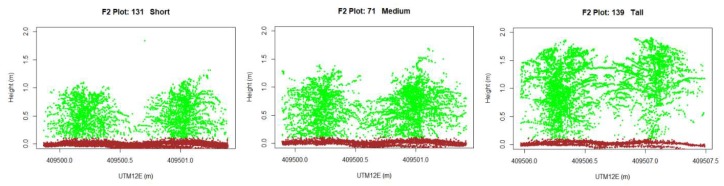
2D visualizations of lidar data. Green colored dots are plant hits and brown colored dots are soil hits. Plots 131 (**left**), 71 (**center**), and 139 (**right**) represent genotypes of small, medium, and tall size maize plants respectively.

The resolution of lidar measurements in the direction of travel is defined by the combination of two variables: the ground platform speed of operation (0.48 m s^−1^), and the frequency of the lidar system data acquisition, which was set at 5 Hz in this study. Due to small fluctuations in travel speed, we estimate that, overall, lidar sweeps occurred every 0.1 m. Thus, every experimental plot was scanned with approximately 28 consecutive lidar sweeps. Figure 8 displays all lidar points produced in three plots of plants with maize genotypes of different canopy sizes and morphology. It is worth noting that canopy morphology affected the proportion of lidar beam hits on the plant (green color dots) over lidar beams that hit the soil (brown color dots) as they travel through openings in the plant canopy. The fraction of lidar beams hitting plant material was computed as 0.211, 0.429, and 0.616 for plots 131 (short), 71 (medium), and 139 (tall plants) respectively.

### 3.4. Application of Lidar Phenotyping to Quantitative Trait Locus (QTL) Identification

To test the utility of the lidar-based CH with manual measurements for identification of quantitative trait locus (QTL), we compared the lidar-based CH with manual measurements of shoot dry mass taken the same week as the final height measurement. CH at 64 DAP had high genetic correlations with shoot dry mass for both well irrigated and drought-stressed treatments (Table 2). The lidar-based CH measurements also gave high estimates of broad-sense heritability compared to that of manual shoot dry mass (Table 2).

**Figure 9:**
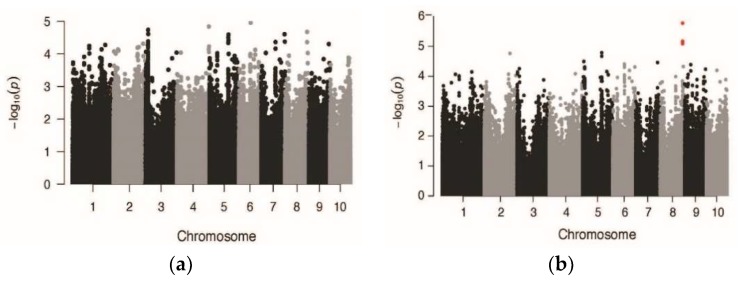
Genome-wide association (GWA) mapping of lidar-based canopy height in well-irrigated (**a**) and drought-stressed (**b**) treatments. Colored symbols highlight the three markers that have the lowest *p*-values. These are located at 161523134, 161525268, and 161525282 on chromosome 8 in the Maize B73 RefGen_v2 genome.

Due to the small size of the plants at the early measurement dates and the correspondingly larger range of plant heights at the last measurement date (Table 1), we used the 64 DAP measurements to scan for QTL. We scanned for QTL associated with phenotypic variation in mean CH in both well-irrigated and drought-stressed treatments using GWA mapping. We used a mixed linear model (MLM) with a kinship matrix (K) and population structure (Q) as covariates [25]. After correction for multiple hypothesis testing, we did not detect any SNPs that were significantly correlated with height in either treatment (Figure 9). This is not surprising considering that, while maize height has been shown in previous studies to be highly heritable, it also is a highly polygenic trait [9].

Although not significant after multiple hypothesis testing correction, the three SNPs with the lowest *p*-values in our analysis were located within the same gene model on chromosome 8 (Figure 9b, highlighted in red). This gene, GRMZM2G371033 (Zm00001d012015), encodes a SQUAMOSA promoter binding protein (SBP)-box transcription factor. Other members of this class of proteins have been shown to be involved in the regulation of reproductive development in maize [27,28]. These SNPs are also within a region containing previously identified QTL for flowering time and plant height traits [9].

## 4. Discussion

There are many challenges in attempting outdoor deployment of electronic systems from moving ground platforms in arid climates. Some of these challenges include ambient temperatures over 40 °C, low-frequency vibrations of the vehicle engine, vehicle stability, and interference from soil particles or dust. In spite of challenging environment, the mechanical and electrical subcomponents of the dual lidar system described in this paper performed with excellent precision. The phenotyping system was reliable and successfully captured plant architectural data of a diverse population arranged in 1728 experimental plots six times over a period of 42 days when the maize plants experienced high growth rates. Moreover, the system hardware design and data processing algorithms were both flexible and robust, allowing efficient lidar data capture from a collection of germplasm of contrasting phenotypes.

The SZ-16D lidar units used in this system were not systematically calibrated to determine absolute accuracy prior to field deployment in this study. The overall performance of the lidar measurement system is based on the intrinsic performance of the sensors coupled with the algorithms built to process the raw data on a relative scale. The results of the pre-season dynamic check on ZREF described in Section 3.1 were used to gauge absolute accuracy of the critical parameter used to estimate canopy height. Although the system check described in Section 2.2.6 was not a rigorous test of accuracy, the comparison between manual and lidar-based measurements of ZREF in dynamic mode were very close and within 10 mm, which is a relatively narrow band of uncertainty. The results from this accuracy check provided the confidence to infer that the output of the lidar system was capable of consistently characterizing canopy height and cover in this experiment. Moreover, when comparing the manual and lidar-based measurements of CH, the RMSE and r^2^ values obtained (0.068 m and 0.81 respectively) provide solid validation for the implementation of this lidar system under the field conditions where the experiment took place. Overall, RMSE in CH estimations during dynamic mode deployment in this study was comparable to experiments carried out under static conditions [21], and in other cases where lidar technology was implemented using high-cost, high-resolution instrumentation and employing algorithms for 3D cloud data post-processing [18,19]. Moreover, the linear regression analysis of lidar-based CW and image-based fractional canopy cover (Figure 6) yielded an r^2^ of 0.73, providing confidence the lidar-based approach is a practical method to estimate this parameter.

There were four innovative data reduction methods employed in the design of the lidar system for this application to reduce the size of the raw dataset from field deployment into sizes that would streamline the post-processing phase and still provide a higher resolution than is possible with a human-based crew: limiting operational laser sweep/FOV to 90°, from 270°; sampling frequency and data acquisition limited to 5 Hz; data acquisition limited to the experimental area of the field; and measurement product extraction (CH and CC) from each 2D sweep as opposed to point cloud analysis and extraction. Data reduction approaches were successfully implemented for efficient same-day post-processing to generate summary by-plot statistics in commonly used file formats. This feature expedited the analytical phases of field phenotyping and maintained high-throughput from end to end. Lidar data accuracy was validated through independent measurements of canopy height and cover in a subset of 75 experimental units. Every experimental plot contained around 6000 lidar points which was of sufficient data density to extract meaningful estimates of canopy height and canopy cover per experimental unit (i.e., plot). Other methods and applications of lidar systems used under indoor conditions reported measurements of 12,000 points per leaf [17], which is arguably excessive, not ready for field deployment, and prohibits high-throughput of the entire system. Although limiting the data acquisition to 5 Hz greatly benefited in reducing raw data sizes for canopy height and canopy cover, it is not necessarily optimal for other measurement products that may be desirable, such as individual leaf width, leaf angle or plant organs. Attempts at 3D lidar data visualizations of point cloud data in the 75 experimental subsets confirmed this to be the case.

Identification of genetic variants that control plant height in maize has been challenging, due to the large number of low effect-size genes affecting this trait [9]. This is a situation similar to that in human height, a classical example of a highly heritable, yet highly polygenic phenotype [29]. Very large population sizes have been shown to improve detection of loci underlying human height variation [30]. It is likely that increased sample sizes will similarly aid in the identification of QTL for maize height. This new lidar height technology will provide the throughput required to greatly increase the size of phenotyped populations. Additionally, the low cost and computational simplicity of this system will allow for deployment in multiple environments [18,19].

## Figures and Tables

**Table 1:** Time series data of per-plot mean canopy height (CH) and canopy cover (CC) values of three genotypes in the maize population included in this study.

DAP	Genotype ID/Morphology Class					
	WIL500/Short		Z022E0104/Medium		SC357/Tall	
	CH-m	CC-%	CH-m	CC-%	CH-m	CC-%
22	0.09 (0.07)	–	0.12 (0.09)	1.5 (8.6)	0.24 (0.10)	13.9 (10.8)
28	0.17 (0.09)	2.8 (8.5)	0.24 (0.11)	6.5 (13.6)	0.40 (0.14)	23.3 (13.8)
34	0.23 (0.10)	6.9 (10.9)	0.29 (0.10)	19.8 (18.9)	0.57 (0.14)	34.7 (12.1)
40	0.27 (0.12)	9.0 (11.5)	0.39 (0.11)	28.3 (18.8)	0.75 (0.25)	41.1 (20.1)
47	0.38 (0.16)	10.2 (11.3)	0.61 (0.12)	45.7 (29.6)	1.01 (0.30)	63.4 (30.9)
64	0.78 (0.31)	18.2 (19.4)	1.11 (0.29)	49.9 (25.0)	1.52 (0.40)	73.7 (27.5)

Numbers in parenthesis indicate value of one standard deviation.

**Table 2:** Comparison of genetic correlations and heritabilities for manual and lidar-based traits.

Treatment	Genetic Correlation between Manual Shoot Dry Mass and Lidar-Based CH	Shoot Dry Mass Heritability (%)	Lidar-Based CH Heritability (%)
Well-irrigated	0.70 *	59.4	78.0
Drought-stressed	0.68 *	42.8	72.7

* *p* < 0.0001.
